# Glucuronidation and UGT isozymes in bladder: new targets for the treatment of uroepithelial carcinomas?

**DOI:** 10.18632/oncotarget.12277

**Published:** 2016-09-27

**Authors:** Vikram L. Sundararaghavan, Puneet Sindhwani, Terry D. Hinds

**Affiliations:** ^1^ Department of Physiology & Pharmacology, Center for Hypertension and Personalized Medicine, University of Toledo College of Medicine, Toledo, OH, USA; ^2^ Department of Urology, University of Toledo College of Medicine, Toledo, OH, USA

**Keywords:** UGT, UDP-G glycosyltransferase, glucuronidation, bladder cancer, nuclear receptors

## Abstract

Bladder cancer has been linked to numerous toxins which can be concentrated in the bladder after being absorbed into the blood and filtered by the kidneys. Excessive carcinogenic load to the bladder urothelium may result in the development of cancer. However, enzymes within the bladder can metabolize carcinogens into substrates that are safer. Importantly, these proteins, namely the UGTs (uridine 5-diphospho-glucuronosyltransferases), have been shown to possibly prevent bladder cancer. Also, studies have shown that the UGT1 expression is decreased in uroepithelial carcinomas, which may allow for the accumulation of carcinogens in the bladder. In this review, we discuss the UGT system and its protective role against bladder cancer, UGT genetic mutations that modulate risk from chemicals and environmental toxins, as well as targeting of the UGT enzymes by nuclear receptors.

## INTRODUCTION

Bladder cancer (BC) is the fourth leading cause of cancer in men and constitutes about 5% of all new cancer cases in the US [1]. The American Cancer Society estimates that in 2016 there will be approximately 76,960 new cases of bladder cancer (58,950 men and 18,010 women) in the United States [1]. Out of these cases, there are an expected 19,390 deaths - 11,820 men and 4,570 women [1].

The bladder is exposed to a wide array of carcinogens that are filtered from the blood. Any carcinogens that are ingested or are otherwise absorbed in the blood could potentially be exposed to the bladder urothelium. The development of bladder cancer may be a result of excessive carcinogenic load to the urothelium, combined with the insufficient metabolism of these carcinogens. Enzymes in the bladder that metabolize carcinogens into safe compounds, such as the uridine 5’-diphospho-glucuronosyltransferases (UGTs), may provide protection against cancer. Genetics, smoking, environmental exposure, age, and gender are all factors that are thought to play a role in the development of bladder cancer. Notably, men have 3-4 times higher lifetime risk of getting bladder cancer than women [1], which may be explained by a difference in sex hormones as will be illustrated later.

Smoking tobacco is a well-studied risk that is associated with bladder cancer. Results from a systematic literature review from 1985-2006, determined that cigarette smoking is the primary reason for bladder cancer and accounts for half of all cases in developed countries [2]. Exposure from the environment and occupation have also been implicated in the pathogenesis of bladder cancer. In a review of case-control studies dating back to 1989, Paolo et al. reported that the proportion of bladder cancers attributable to occupation ranges between 0-2% and 24% [3]. The reason for this broad range seems to be due to the amount of exposure to bladder carcinogens [3]. Disinfection of drinking water via chlorination forms byproducts including trihalomethanes such as chloroform, bromodichloromethane, chlorodibromomethane and bromoform [4]. These chlorinated byproducts have been shown to be carcinogenic in several animal studies [5–7]. A meta-analysis containing six case-control studies (6084 patients with bladder cancer and 10,816 control) and two cohort studies (124 patients with bladder cancer), determined that long-term consumption of chlorinated drinking water is linked to bladder cancer, particularly in men [8]. This meta-analysis reported the increased risk of bladder cancer with the combined odds ratio (OR) of 1.4 for men and 1.2 for women [8]. Finally, several source water contaminants such as arsenic, asbestos, radon, agricultural chemicals and hazardous waste have also been studied to determine if they are carcinogens [9]. Arsenic is one of the contaminants that has the strongest associations with bladder cancer [10]. Chen et al. observed that residents in an area with high levels of arsenic in their drinking water experienced significantly greater levels of bladder cancer as well as other forms of cancer [10]. UGTs are thought to play a fundamental role in the metabolism of bladder carcinogens and may function to prevent the harmful effects of toxins on the bladder.

**Figure 1:**
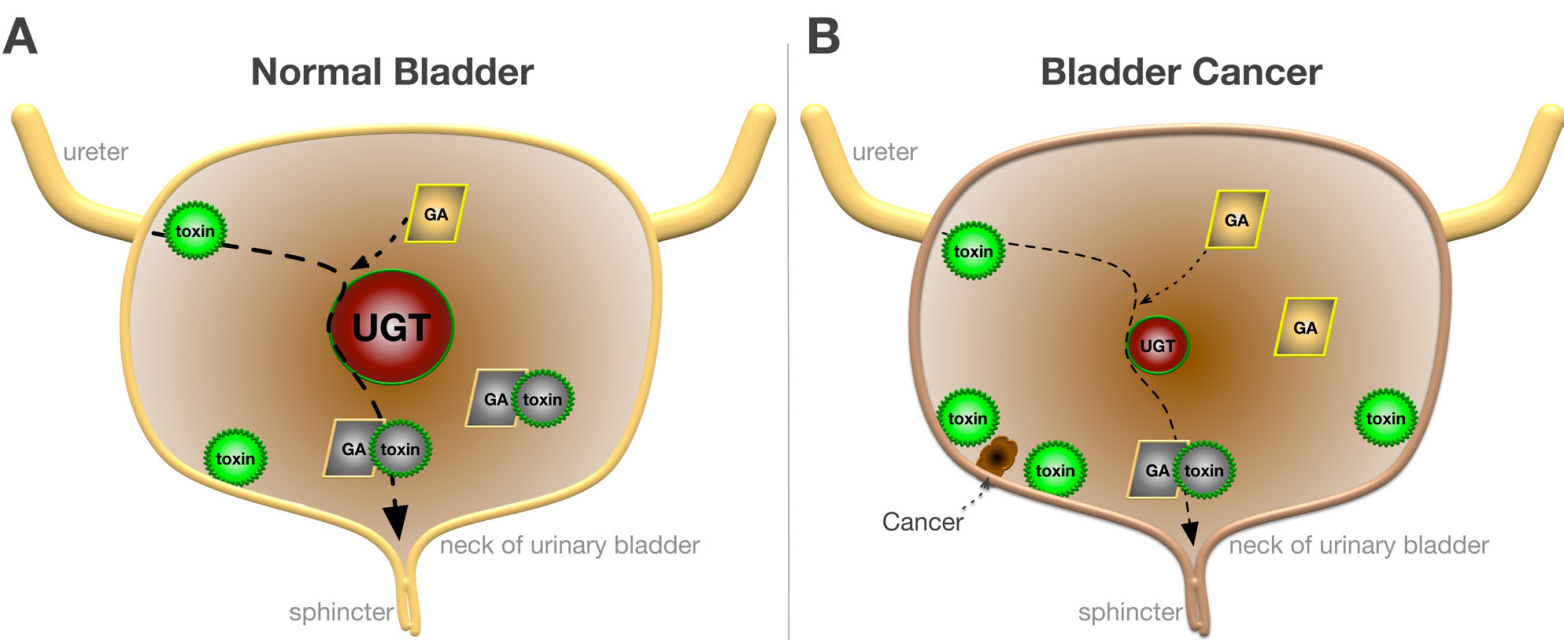
Function of UGT in Normal and Bladder Cancer A. In normal bladder toxins (green) undergo glucuronidation by UGTs (red) with glucuronic acid (GA) to form safer metabolites (gray) that are excreted. B. In bladder cancer glucuronidation of toxins are reduced due to the lower expression of UGTs, which causes an accumulation of toxins and development of uroepithelial carcinomas.

UGTs are enzymes which catalyze the glucuronidation of several exogenous and endogenous compounds (Figure 1) [11]. Endogenous substrates include bile acids, thyroid and steroid hormones, bilirubin and dietary products; while exogenous substrates encompass thousands of xenobiotics including carcinogens, therapeutic drugs, and environmental pollutants [11]. UGTs function in the metabolism of exogenous substrates is thought to play an inhibitory role in the development of bladder cancer. Studies in both mice and human bladder cancer cell lines suggest an inverse relationship between the amount of UGT expressed and the presence of bladder cancer. Substrates that increase the expression of UGT could provide novel therapeutic advances in the treatment of bladder cancer. Herein, we will discuss the role of the UGT isozymes in bladder carcinogenesis, and propose therapeutic strategies based on modulation for further study.

## UGT SUPERFAMLIY

**Figure 2:**
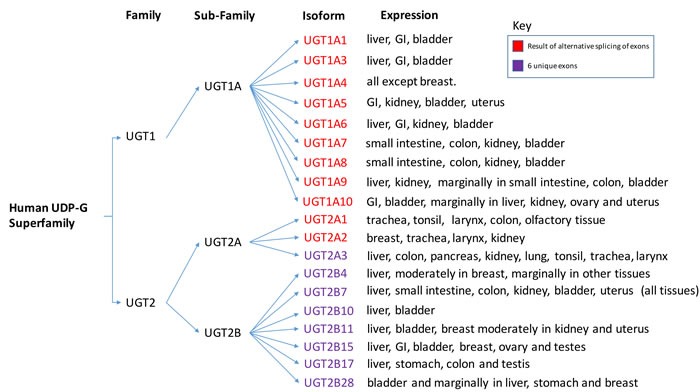
UGT Superfamily The different isoforms of the human UGT superfamily based upon differential splicing of the RNA transcripts. Additionally, tissue expression data of the different isoforms are listed. Psueudogenes are not included. Pleases note that information for this diagram was obtained from the following [11, 13] and modified into a graphical representation.

The 117 members of the mammalian UGT superfamily are divided into four families: UGT1, UGT2, UGT3 and UGT8 [12]. Humans only express UGT1 and UGT2, which are further organized into three subfamilies: UGT1A, UGT2A and UGT2B (Figure 2) [11]. The three human subfamilies listed, give rise to 16 different isoforms. While most of the isoforms are expressed in the liver, they are also seen in various other human tissues, including the urinary bladder. The human UGT1 gene family is located on chromosome 2q37, while the UGT2 family are located on chromosome 4q13 [12]. The UTG1 gene is approximately 200 kb in size and contains shared exons 2-5 and 13 unique promoters/first exons. This results in 13 potential transcripts that can be made at the UGT1 gene locus, however, 4 of the 13 first exons are labeled as pseudogenes and contain mutations [12]. Similar to the UGT1A enzymes, UGT2A1 and 2A2 have a unique first exon with a shared set of 5 downstream exons [12]. The resulting proteins have identical C-terminals with a distinctive N-terminal due to alternative splicing of the first exon with downstream shared exons. In comparison, the UGT2A3 gene has six exons that are not shared with UGT2A1 or 2A2. Similar to UGT2A3, the six exons of UGT2B do not use alternative splicing as seen in the rest of the human UGT superfamily [12]. The diversity of the UGT isoforms may function to control specific aspects of detoxifications. Further investigation is needed to determine the roles of the UGT isozymes and their specificity.

## UGT FUNCTION

UGTs are phase II drug metabolism enzymes that act as a co-substrate in the formation of lipophilic glucuronides, primarily in the liver, but also in various extra-hepatic tissues [11]. Glucuronidation, performed by UGTs, is a type of conjugation reaction in which the membrane-bound UGT, catalyzes the transfer of the glucuronic acid moiety from UDP-glucuronic acid, the co-substrate, to the substrate. The resulting glucuronide, or conjugated substrate, is much more hydrophilic than before. The glucuronide can now be excreted via biliary or renal routes, which confers the anti-toxic effects to this pathway. There have been over 350 compounds that have been identified as substrates for the UGT superfamily [13]. UGTs have roles in several biological processes, including phase II drug metabolism, and bilirubin conjugation. Polymorphisms of the Ugt1 gene locus has long been linked with both Crigler-Najjar and Gilbert's syndrome which causes a decrease in bilirubin conjugation and an elevation of free bilirubin in serum [14]. Crigler-Najjar syndrome results in extremely high levels of unconjugated bilirubin requiring liver transplantation and may lead to death. Patients with Gilbert's syndrome, however, have only slightly elevated levels of bilirubin, but, and interestingly, have reduced incidences of obesity, type II diabetes, and cardiovascular disease [14]. It has recently been shown that moderately increasing serum bilirubin levels activate the nuclear receptor PPARα to inhibit lipid accumulation and lower blood glucose [15]. A global knockout of the Ugt1 locus in mice causes an extreme level of unconjugated bilirubin in serum that is comparable to the levels in patients with Crigler-Najjar type 1 disease [16]. Furthermore, the UGT1 global knockout in mice is fatal in neonates with death in two weeks following birth [16]. Therefore, bladder cancer studies in mice with UGT1 knocked out have not been performed. A tissue-specific knockout of the Ugt1 locus in the bladder may be of value in understanding the role for the isozymes. However, this is yet to be done. More recently, small nucleotide polymorphisms (SNPs) of the UGT isozymes have been identified as both protective and as a risk factor for bladder cancer [17–19], which will be discussed further below.

## ROLE OF UGT IN BLADDER CANCER

UGTs may be inhibitory to the development of bladder cancer, as was demonstrated by two different types of studies: one that measured the expression of UGTs in tumor vs. normal bladder tissue, and another that analyzed the effects of various UGT SNPs on expression and bladder cancer.

UGTs are highly expressed on the surface of normal bladder tissue. However, expression was shown to be significantly reduced in malignant tissue. Giuliani et al. conducted a study using a polyclonal antiserum that targets UGT1A isoforms and analyzed bladder cancer samples with immunochemistry [20]. The results of this study varied based on the type of bladder cancer. They found that while 2/2 papillomas and 8/11 papillary bladder carcinomas showed UGT expression, 3/11 papillary and 3/6 urothelial carcinomas were essentially UGT-negative [20]. Based on the results from immunochemistry, the authors concluded that UGT1A down-regulation is an event that occurs during chemical carcinogenesis [20]. Furthermore, the data suggests that one group of invasive bladder cancer cells may maintain the UGT expression while the other group lacked UGT expression [20]. In another study by Guiliani et al., ten bladder transitional cell carcinomas where analyzed along with normal bladder tissue using RT-PCR, and it was determined that while UGT was expressed in all of the normal bladder tissue samples, it was completely absent in 4/10 tumor samples, lowly expressed in 5/10 samples and normally expressed in only one sample [21]. The results from both studies by Guiliani et al. were further validated by an immunochemistry analysis of the expression of UGT1A in 145 bladder tumor specimen's verses 101 benign bladder tissues samples [17]. In benign urothelial tissue, UGT1A was detected in 100/101 specimens, compared with 130/145 neoplastic urothelial samples [17]. Overall, UGT1A was found to be substantially reduced in tumors when compared to benign tissue (P < 0.001) [17]. Specifically shown is a strong association between lower UGT1A expression and recurrence of high-grade non-muscle-invasive tumors (P = 0.038) [17]. Finally, multivariate analysis demonstrated that the loss of UGT1A expression was an independent prognosticator for disease-specific mortality in patients with muscle invasive tumors (P = 0.010) [17].

Furthermore, the grade of the tumor was shown to be correlated with UGT1A expression, with high-grade bladder carcinomas showing significantly less expression as compared with low grade and papillary urothelial neoplasm of low malignant potential (PUNLMPs) (P < 0.001) [17]. Therefore, UGT1A may prevent both bladder carcinogenesis and tumor progression, and reduced expression may allow for the accumulation of more toxins (Figure 1).

## UGT MUTATIONS ENHANCE BLADDER CANCER RISK FROM SMOKING

Smoking has long been determined to be a significant risk factor for the development of bladder cancer. Also, risk has been studied in conjunction with genetic variants of the UGT isozymes, as well as other loci. In a study of 282 Japanese patients with transitional cell carcinoma and 257 healthy controls, it was determined that the combination of UGT2B7*1/*2 or *2/*2 with N-acetyltransferase 2 (NAT2) genotypes showed the highest risk for bladder cancer (OR = 4.2) after being adjusted for age and gender in these patients [22]. Interestingly, they determined that smoking status did not appear to be a risk for bladder cancer [22]. However, this conclusion contradicts a European study of 3,942 cases and 5,680 controls which determined that common genetic polymorphisms modify the effect of smoking on the risk of developing bladder cancer [23]. When analyzing 12 susceptible loci in relation to bladder cancer, two most notably showed significant additive gene-environment interactions: NAT2 (P = 7×10-4) and UGT1A6 (P = 8×10-4). The authors conclude that the number of bladder cancer cases prevented due to smoking elimination is larger for individuals with high genetic risk [23]. Based on these studies, UGTs seem to play an important role in metabolizing carcinogens, particularly those from smoking. Carcinogen metabolism appears to occur in the bladder, but also in other tissues prior to them being filtered into the bladder, particularly within the UGT2 family of isoforms. RT-PCR results in a study by Bushey et al. showed that UGT2A1 expression distribution was highest in the trachea followed by the tonsil and larynx [24]. These areas, which experience direct tobacco insults, mainly from polycyclic aromatic hydrocarbons (PAH), utilize UGT2 isozymes to neutralize these carcinogens. Tobacco-related throat cancers seem to be regulated by the UGT2 gene locus, which may suggest a predilection for mutations in these isozymes in these carcinomas. UGT1 isoforms may have more of a role in bladder cancer detoxification. However, the functions of UGT1 or UGT2 and tissue-specific responses need to be investigated.

## SNPS IN THE UGTS MODULATE BLADDER CANCER RISK

Carcinogens may cause down-regulation of the UGTs. Conversely, genetic variations in the UGT gene locus could account for different levels of UGT isozyme expression, which may promote or protect from bladder cancer. A genome-wide association study of bladder cancer that analyzed 12,254 individuals with bladder cancer and 53,395 controls uncovered three genomic regions associated with bladder cancer including a genetic variant of the UGT1A gene locus, rs11892031 (P = 1.0×10^-7; OR per C allele = .84, 95% CI = 0.79-0.89) [18]. Therefore, people with the genetic variant rs11892031 may have a greater risk of developing bladder cancer [18]. To further examine the association of rs11892031 with bladder cancer, Selinski et al. analyzed eight case-control studies including 1,805 cases and 2,141 controls from the IfADo UBC study group and further supplemented this data with a meta-analysis of all published data, including 13,395 cases and 54,876 controls [25]. The results of this analysis confirmed the genome-wide association study (GWAS) by Rothman, which identified the association between rs11892031 and bladder cancer [25]. The GWAS of bladder cancer prompted further fine mapping of the UGT1A locus, which allowed Tang et al. to identify a SNP rs17863783 [19]. This variant was found to accompany increased mRNA expression of UGT1A6.1 protein, a splicing mRNA isoform of UGT1A6 [19]. Due to increased expression of UGT1A6.1, the SNP rs17863783 was concluded to be protective against bladder cancer [19]. In another analysis of the UGT gene locus, Wang et al. analyzed 75 SNPs that were identified from the HapMap database ( http://www.hapmap.org) [26]. When considering 1501 subjects (718 cases, 783 controls), eighteen SNPs in the UGT gene family were identified to be associated with bladder cancer [26]. Sixteen of the eighteen SNPs are located in the UGT1 family, and two are of the UGT2 family [26]. When adjusting for multiple comparisons, eleven SNPs remained significant. SNP rs7571337 was found to be most protective against bladder cancer, showing a 29% decrease bladder cancer risk (OR = .71, 95% CI = 0.56-0.90) [26].

Bladder cancer is normally the result of environmental insults combined with genetic mutations. Benzidine has been used as a reagent base for the production of copious dyes, cotton, and leather since the 1850s [27]. Studies have shown the detrimental effects benzidine exposure on workers and the International Agency for Research of Cancer (IARC) lists benzidine as a proven Group I bladder carcinogen in humans [28]. A study by Lin et al. analyzed the UGT2B7 gene locus for benzidine-exposed Chinese subjects [29]. Their data showed that a UGT2B7 polymorphism (T/T genotype) was much more prevalent in the Chinese population than for Caucasians, and this SNP was found in a higher prevalence of bladder cancer cases compared with healthy controls (25 vs. 9% OR) [29]. Ciotti et al. analyzed five different UGT enzymes for its ability to metabolize benzidine and its metabolites [30]. Overall, UGT1A9 had the highest overall rate of metabolism, followed by UGT1A9 > UGT1A4 > > UGT2B7 > UGT1A6 = UGT1A1 [30]. However, these enzymes showed a high degree of substrate specificity, with some metabolites being very poorly broken down by certain isoforms [30]. Altogether, genetic anomalies in the UGT isozymes and exposure to toxins lead to the development of bladder cancer.

## UDP EXPRESSION AND FLUID INTAKE IN BLADDER CANCER

Fluid intake has been considered with respect to genetic variants of the UGT isozymes and bladder cancer. A study looking at total and specific fluid intake in relation to bladder first concluded that high total fluid intake (OR = 2.02 for > = 2789 ml/day), soda intake (OR = 1.86 for > = 0.71 servings/day) and decaffeinated coffee (OR = 2.26 for > = 2 servings/day) are risk factors for bladder cancer; while tea (OR = .62 for > = 0.71 servings/day) and moderate alcohol consumption (OR = 0.72 for 0.1-0.38 servings of wine/liquor) are protective [26]. When this data was stratified against various genotypes of the UGT SNP rs7571337, it was concluded that the increased risk presented by total fluid intake, soda intake, and coffee consumption were significant only among the rs7571337 AA genotype, while the inverse relationship seen with tea and alcohol were only observed among the AG/GG carriers [26]. This further supports the notion that the genetic makeup of the UGT gene locus can play a key role in the prognosis and development of bladder cancer.

## NUCLEAR RECEPTOR REGULATION OF UGT EXPRESSION IN BLADDER CANCER

**Figure 3:**
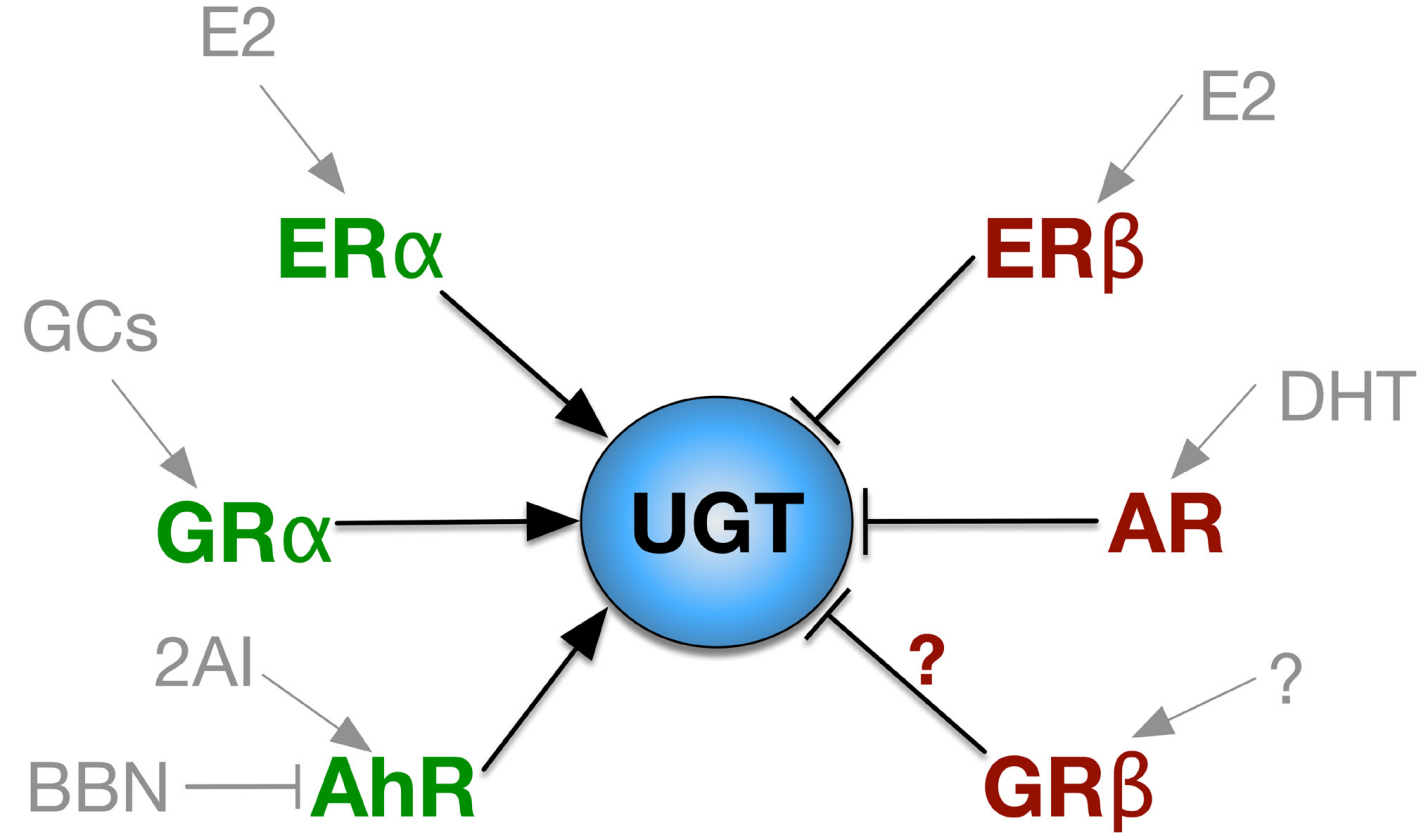
Nuclear Receptor Regulation of UGT Nuclear receptors that increase UGT expression are labeled in green and in red for inhibitory receptors. The ligands of each nuclear receptor is labeled in gray. E2, estradiol; GCs, glucocorticoids; BBN, N-butyl-N-(4-hydroxybutyl)nitrosamine; 2AI, 2,2’-aminophenyl indole; DHT, dihydrotestosterone.

Since UGTs may play a significant role in the pathogenesis of bladder cancer, it is important to determine what modulates expression as this could provide therapeutic alternatives. A study by Lida et al. used a N-butyl-N-(4-hydroxybutyl)nitrosamine (BBN) induced mouse urinary bladder carcinogenesis model to test the effect of the aryl hydrocarbon receptor (AhR) on UGT1A modulation [31]. BBN was found to irreversibly down-regulate basal UGT1A mRNA expression after four weeks of treatment. Expression of UGT1A mRNA was much lower in the AhR KO mice and decreased in the bladder of wild-type mice, as well as its’ regulated gene Cyp1a1 [31]. AhR binds to the promoter of both of these genes and is lower in bladder cancer, indicating a role of AhR in the protection of uroepithelial carcinomas. Additionally, AhR KO mice did not display BBN-induced downregulation of UGT1A or Cyp1a1, which was down-regulated by BBN-induced bladder cancer of wild-type mice. Altogether, these results suggest that in mice with BBN-induced bladder carcinogenesis, UGT1A mRNA is repressed through the reduction of the AhR signaling pathway (Figure 3) [31]. Compounds such as such as 2,2’-aminophenyl indole (2AI), a potent synthetic agonist of AhR [32], may help to protect against bladder cancer by increasing UGT isozyme expression. Additional AhR ligands, such as those tested in breast cancer, including 4-hydroxtamoxifen, mexiletine, nimodipine, omeprazole, sulindac and tranilast [33] should also be investigated for protection against uroepithelial carcinomas.

UGT regulation also occurs via hormones such as androgens and estrogen. The explanation for the increased prevalence of bladder cancer in males has been poorly understood. Although bladder cancer is not considered to be dependent on hormone activity, sex hormones seem to play a role in regulating the expression of UGT1A. Izumi et al. found that UGT1A expression was positively correlated with the estrogen receptor α (ERα) while being negatively correlated with the estrogen receptor β (ERβ) in bladder specimens [17]. Additionally, 17β-Estradiol increased UGT1A expression in normal urothelium, while it decreased UGT1A expression in bladder cancer cell lines. Both ER isoforms can bind to 17β-Estradiol (Figure 3), which may indicate a differential function in bladder cancer development. Most likely, ERα is reduced in uroepithelial carcinomas and ERβ is increased. Furthermore, down-regulation of the UGT1A subtypes was observed in mice with ovariectomy [17]. More studies on the ER isoforms and their involvement in bladder cancer development are needed. The lower incidence of bladder cancer in females may be through increased UGT isozyme activity and detoxification of the bladder. However, this is yet to be investigated.

Androgens have also been studied to determine their role in bladder cancer progression and studies suggest they may have an impact, or at least the androgen receptor (AR). To test this idea, Miyamoto et al. treated the following groups with BBN: wild-type male and female mice, with and without castration of male mice, AR knockout (ARKO) female and male mice, and with and without DHT supplementation in male mice [34]. Gender differences were noticed as expected. Over 92% of wild-type males treated with BBN developed cancer whereas only 42% of wildtype females developed bladder cancer [34]. Notably, none of the ARKO mice, male or female, developed bladder cancer [34]. Twenty-five percent of ARKO mice treated with BBN and supplemented with DHT developed bladder cancer, as well as 50% of castrated wild-type male mice [34]. DHT treatment of SVHUC-AR (androgen receptor) cells showed a 19-75% decrease in UGT1A expression compared to the mock treatment [35]. With respect to individual isoforms, DHT treatment reduced the levels of UGT1A1, UGT1A4, and UGT1A9 by 31%, 31% and 63% respectively [35]. Importantly, Kashiwagi et. al. showed that AR activation induces chemoresistance in bladder cancer, possibly by modulating NF-κB [36]. These findings support the idea that both androgens and AR are possibly involved in bladder cancer, which may be linked to repression of the UGT isozymes.

Glucocorticoids (GCs), such as dexamethasone (Dex), may play a role in the regulation of UGT isozyme expression in the bladder. We have recently shown that Dex reduced migration of human bladder cancer cells [37]. GCs bind and activate the ligand-binding GC receptor (GR), GRα [37–41]. There are at least five isoforms of the GR that exist due to alternative splicing [39]. The GRβ isoform lacks the ligand-binding domain for GCs [39, 41], and has been shown to be inhibitory to GRα [40–44]. Importantly, GRβ increases migration of human bladder cancer cells [37], possibly through inhibition of GC-induced GRα activity [45]. GRβ inhibits GCs by binding to the GRα isoform and suppressing gene activity [37, 38, 40, 41, 45, 39]. A higher total GR expression has been correlated with a better prognosis in bladder cancer [45, 46, 47]. However, the specific roles of GRα or GRβ in bladder cancer are unknown. Interestingly, Jemnitz et al. reported an increase in UGT1A1 expression when mice hepatocytes were exposed to clofibrate (Cl) and Dex [48]. Additionally, they determined that 3-methylcholanthrene (MC) increased the concentration of UGT1A6 [48]. While MC is a carcinogen, safe compounds that increase UGT expression may be therapeutic. Sugatani et al. also reported increased expression of the UGT1A1 isozyme after GC treatment, however in human hepatic cell lines [49]. They propose that GR activation enhances constitutive androstane receptor / pregnane X receptor (CAR/PXR) mediated UGT1A1 regulation [49]. The involvement of the GRα isoform should be investigated in bladder cancer cell lines to determine whether upregulation of the UGT isozymes occurs, or if the GRβ inhibits expression to lead to uroepithelial carcinomas. Targeting of the human GRβ with an anti-molecules, such as Sweet-P [37, 50], may cause a rise in the UGT isozyme expression, especially since GCs have been shown to increase expression in liver. The role of GCs and GR isoforms, as well as their involvement in regulating UGT expression in the bladder, is needed.

Targeting of AR, ERβ or GRβ may provide a novel treatment for bladder cancer. GRα, AhR, and ERα ligands may be beneficial in the treatment of uroepithelial carcinomas. The mechanism behind these findings may be explained, at least in part, by nuclear receptor regulation of UGT expression in bladder and other tissues. However, more studies are needed to uncover their roles in uroepithelial carcinomas.

## CONCLUSIONS

UGTs are enzymes that act as potent metabolizers of carcinogens and may protect the bladder from the accumulation of toxins. UGT isozymes have been shown to be reduced in several cancers. UGT down-regulation, particularly UGT1A, appears to occur in bladder carcinogenesis. Novel methods of targeting UGT1A may serve as a potential therapeutic for uroepithelial carcinomas. Another approach may be the exogenous introduction of particular UGT isoforms, for example, UGT1A, into the bladder. While this review analyzes several SNP studies and GWAS, experiments using Ugt1 tissue-specific knockout models, such as in the bladder, should be conducted to further understand the impact of the UGT enzymes in uroepithelial carcinomas. Lastly, further research should be carried out to determine whether ligands acting on sex hormone receptors (AR or ER) and the GR isoforms or AhR targeting can provide preventive measures against bladder cancer as these are known modifiers of the UGT isozymes. Sensitization to GCs by inhibition of the GRβ isoform by Sweet-P may provide an avenue for increasing the UGT isozymes in the bladder. Increasing the UGT isozymes in the bladder may prove advantageous to therapeutics.
